# Responsive *Feeding* Environments in Childcare Settings: A Scoping Review of the Factors Influencing Implementation and Sustainability

**DOI:** 10.3390/ijerph191911870

**Published:** 2022-09-20

**Authors:** Jessie-Lee D. McIsaac, Madison MacQuarrie, Rachel Barich, Sarah Morris, Joan C. Turner, Melissa D. Rossiter

**Affiliations:** 1 Department of Child and Youth Study, Faculty of Education, Mount Saint Vincent University, Halifax, NS B3M 2J6, Canada; 2 Department of Applied Human Sciences, University of Prince Edward Island, Charlottetown, PE C1A 4P3, Canada

**Keywords:** early childhood, responsive feeding, nutrition, implementation, behavior change

## Abstract

Children benefit from responsive feeding environments, where their internal signals of hunger and satiety are recognized and met with prompt, emotionally supportive and developmentally appropriate responses. Although there is existing research on responsive feeding environments in childcare, there is little synthesized literature on the implementation practices using a behavior change framework. This scoping review sought to explore the factors influencing the implementation and sustainability of responsive feeding interventions in the childcare environment, using the behavior change wheel (BCW). A total of 3197 articles were independently reviewed and 39 met the inclusion criteria. A thematic analysis identified the factors influencing the implementation and sustainability of responsive feeding, including the following: (1) pre-existing nutrition policies, (2) education and training, (3) provider beliefs and confidence, (4) partnership development and stakeholder engagement and (5) resource availability. The most common BCW intervention functions were education (n = 39), training (n = 38), environmental restructuring (n = 38) and enablement (n = 36). The most common policy categories included guidelines (n = 39), service provision (n = 38) and environmental/social planning (n = 38). The current literature suggests that broader policies are important for responsive feeding, along with local partnerships, training and resources, to increase confidence and efficacy among educators. Future research should consider how the use of a BCW framework may help to address the barriers to implementation and sustainability.

## 1. Introduction

Establishing early healthy eating behaviors is important for optimal child development and long-term health and wellbeing. Although there is a gap in the available evidence on nutrition intakes for young children (ages 0–5), approximately one in five children (aged 1–8 years) have energy intakes that exceed their energy needs [1]. An energy imbalance and poor eating patterns that are developed early in life are of concern as they can persist through the lifecycle and are associated with chronic diseases in adulthood [2,3,4,5,6]. An adequate intake of healthy food is essential for young children’s growth, however, focusing on food alone is not enough [7,8,9]. Rather, children benefit from responsive feeding environments, where their internal signals of hunger and satiety are recognized and met with prompt, emotionally supportive and developmentally appropriate responses [10]. A responsive feeding environment acknowledges that feeding practices are impacted by food environments and seeks to promote healthy eating as both a practice (e.g., responsive feeding) and a product (e.g., healthy and nutritious foods). There is a strong focus on respectful and reciprocal relationships between the care provider and the child, with age-appropriate responses to hunger cues and satiety [10,11,12].

Developing healthy food behaviors requires the intentional involvement of key caregivers and the practice of responsive feeding encourages responses to children’s hunger cues and encouraging the child’s autonomy in feeding [10]. An essential component of responsive feeding environments centers on the division of responsibility, which encourages caregivers to provide leadership on “the what, when and where” and letting children guide “how much and whether” to eat [13]. Implementing this practice alongside of the provision of consistent and structured family-style meals and snacks (eating with others and choosing your own portions) is suggested to help children become competent eaters with positive food attitudes, food acceptance, self-regulation and skills for managing food contexts [13]. Other research has characterized responsive feeding through a number of key practices related to the feeding environment and to responsive feeding, specifically, such as praising children for trying new foods, asking children about their hunger/fullness and avoiding the use of food rewards [14,15,16]. Other important elements of a responsive food environment focus on role modeling through eating the same food and engagement in conversation during meals [17].

**Figure 1:**
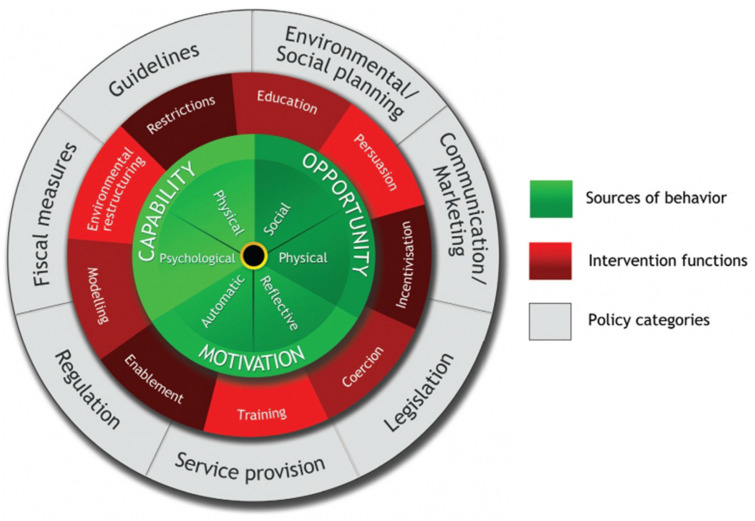
The Behavior Change Wheel [23].

Establishing and sustaining responsive feeding can also be challenging due to the wide range of cultural and social beliefs around feeding young children [18,19], as well as the competing demands on caregivers for time and resources [10]. Each caregiver has their own set of values, beliefs and experiences around food and eating practices, which may influence their actions that control children’s food and portions, deciding the sequence in which food may be eaten, and other actions that may result in a child over or undereating [18,20]. As a result, greater attention is needed to support the implementation and sustainability of responsive feeding practices to bridge research and practice [21]. Research suggests that the most effective interventions to change behavior, including those that relate to responsive feeding, are those that simultaneously and consistently target population, community and individual levels [22]. One established theoretical framework for nutrition-related contexts is the behavior change wheel (BCW) [23], which offers a step-by-step method for multilayered behavior change interventions by selecting the intervention functions and policy categories, which will systematically target multiple levels of behavior to produce the desired outcomes [23]. Within the BCW framework (see Figure 1), the intervention functions are the activities designed to change behaviors and the policy categories are the decisions made by governing authorities related to the interventions.

The COM-B (Capability Opportunity Motivation—Behavior) model is at the center of the BCW to support an understanding of the target behavior, through a consideration of one’s capability to perform the behavior, the opportunity for the behavior to occur and the motivation to perform the behavior [24]. While the BCW has been used in some nutrition-related contexts [25,26,27,28], it is underutilized in an early childhood context and could offer a powerful framework for designing, evaluating and implementing successful interventions for responsive feeding.

While parents have a strong influence on the development of healthy eating behaviors [29], many young children also spend a significant amount of time in childcare [30], which makes this setting an important area to study, given its potential to influence a larger population of children. A recent umbrella review examined the existing interventions to promote healthy eating in childcare environments, with a focus on children’s dietary intake and health outcomes [31]. Although there is existing research that has studied the practice of responsive feeding environments in childcare, there is little synthesized literature on implementation practices using a behavior change framework. Therefore, this scoping review sought to determine what was known about the factors influencing the implementation and sustainability of responsive feeding environments in childcare settings for young children. We explored how studies related to behavior change theory to inform future intervention design and delivery.

## 2. Methods

Scoping reviews provide an opportunity to explore the range of research activities in a particular area and explore gaps that may exist [32,33]. Given the vast amount of literature in the area of early childhood nutrition, but limited synthesis of research in the area of implementation practices for responsive feeding in childcare environments [31], the scoping review methodology was deemed appropriate. We used Arskey and O’Malley’s five stage methodological framework to conduct our review, which includes the follows: (1) identifying the research question, (2) identifying relevant studies, (3) study selection, (4) charting the data and (5) collating, summarizing and reporting the results [32]. The following methods section outlines the methodological steps involved in our scoping review (steps 1–4) and the results section presents the findings from the review.

### 2.1. Identifying the Review Question

This review sought to respond to the following question: “What is known about the factors influencing the implementation and sustainability of responsive food environments in childcare settings?” For the purpose of this review, we characterized responsive feeding environments as those where children’s internal signals of hunger and satiety are recognized by their caregivers and met with prompt, supportive and developmentally appropriate responses [10]. Examples of responsive feeding practices are serving family-style meals and snacks (eating with others and choosing your own portions) [13], acknowledging children for trying new foods, asking children about hunger/fullness and avoiding the use of food rewards [14,15,16]. We defined childcare settings as center or family-based childcare (e.g., organized care that is provided in a physical center or from an individual’s home).

### 2.2. Identifying Relevant Studies

In consultation with a university library subject specialist, an initial search strategy was developed using the electronic databases, Academic Search Premier, Child Development & Adolescent Studies, CINAHL Plus, Education Research Complete, ERIC, MEDLINE, SocINDEX and Google Scholar. The database search included a keyword strategy based on key concepts and using the search function “AND” to identify articles that related to the following: (1) childcare environments, (2) responsive feeding and (3) implementation. Variations for each concept were combined with the “OR” operation to maximize results (e.g., daycare, day care, childcare center, preschool, healthy eating, healthy diet, healthy nutrition, implement*, policy* and standard, guideline). The search was developed and tested using key target articles to ensure sensitivity of the search strategy. The final search for peer-reviewed literature using the above-mentioned databases was performed in February 2021.

We used an iterative process to develop the inclusion and exclusion criteria throughout the review as the team gained a better understanding of the range of literature. For example, we narrowed the focus of our review to upper–middle-income countries [34] to provide greater transferable information to our Canadian research team. Our final inclusion and exclusion criteria are presented in Table 1.

Briefly, we were interested in English language peer-reviewed literature between 2009–2021, with a focus on early childhood (defined as 0–6 years old). Another critical component of the included literature was the focus on implementation and/or the sustainability of population-level responsive feeding practices—not solely the impact of children’s nutrition on health outcomes or specific population groups (e.g., children with identified developmental needs). In particular, we were interested in understanding the implementation of the intervention (including activities, actions and strategies) to support responsive feeding practices in childcare environments. We excluded protocol papers and review studies; however, we searched the corresponding reference lists or subsequent literature to include the finalized study, if available. Following the search, duplicates were removed prior to reviewing and assessing to determine the fit with our inclusion criteria. Two team members independently reviewed abstracts first, followed by the full text of the remaining articles. Where there was disagreement or the need for further discussion, additional reviewers and the lead author were consulted.

### 2.3. Data Abstraction and Synthesis

The final articles included in the review were charted independently by two reviewers and then reviewed and refined in consultation with the primary author. The numerical analysis of the studies [33] provided a summary of the key characteristics of the included studies, according to the country of origin, the theories and frameworks and the research design (see Additional File). Open coding was applied independently by two research team members to ensure rigor in the analysis process and identify common implementation factors in the included studies; patterns in the codes were discussed with the broader team to generate the main themes. The synthesis of the literature also explored whether the included articles contained components of the BCW in the intervention, specifically the *Intervention Functions* and *Policy Categories* of the BCW. Two reviewers independently read through each included study and determined the alignment with elements of the behavior change wheel (Figure 1), indicating if it included (“Yes”) or did not include (“No”) each individual intervention function (activities designed to change behaviors) or policy category (decisions made by governing authorities related to the interventions), or whether there was not sufficient information to categorize (“uncertain”). A thematic analysis [35] was applied to identify the factors influencing the implementation and sustainability of responsive food environments in childcare settings, through identifying, analyzing and reporting patterns within the included studies.

## 3. Results

**Figure 2:**
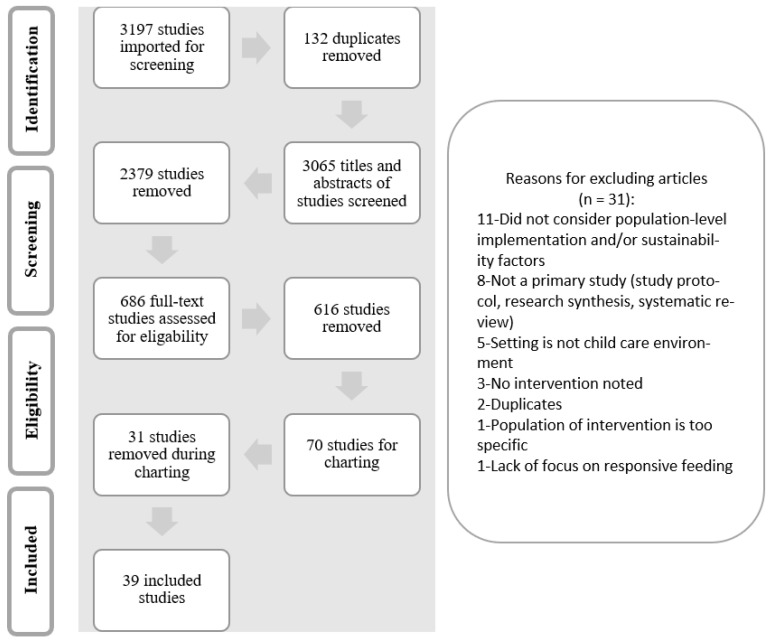
PRISMA Chart of Search Results.

A total of 3197 peer-reviewed articles were retrieved from the search strategy applied for this scoping review. After 132 duplicates were removed, 3065 articles remained for title and abstract screening. Of these, 686 remained for full-text screening. Following full- text screening, 70 articles were left for charting. During the charting process, 31 additional articles were removed as it was determined that they had not met the inclusion and exclusion criteria. The final flow chart diagram, in Figure 2 and Table S1 presents the results from the 39 included articles.

Of the 39 included articles, 31 interventions were from the United States, four were from Australia, one was from Germany, one was from England, one was from Norway and one was from Ireland. One of the included articles presented results from two intervention studies that both used a cluster-randomized controlled trial. When exploring the research designs used in the articles, 29 noted using quantitative data collection (i.e., randomized control trials, n = 10; pre-post evaluation, n = 7; cross sectional, n = 6; prospective observational, n = 1; descriptive analysis of policies, n = 1; other comparison design, n = 2; longitudinal, n = 1; and post-test only, n = 1), five noted qualitative data collection (i.e., interviews and focus groups, n = 1; interviews, n = 3; and focus groups, n = 1) and two noted using a mixed-methods design (i.e., various types of evaluations). Theoretical frameworks and models were noted in 18 of the 39 included articles, with social cognitive theory and the socioecological framework mentioned in multiple articles, whereas others were referenced less often. Some of the ways that settings were described in the articles included licensed childcare centres, day homes, preschools, full-day or half-day programs, school-based, Head Start, Child and Adult Care Food Program (CACFP), private, for profit and non-profit. These intervention settings served children aged zero to six years old, with the majority (n = 17) focused on the ages between two and five years old. These descriptions were not always mutually exclusive from one another as a center could be classified as multiple of the above descriptions (e.g., licensed and Head Start).

The BCW theory [23] was noted in only one of the studies [36] but the intervention description included components that could be considered as a type of intervention function and policy category, with many studies applying multicomponent interventions (see Table 2).

The most commonly found *intervention functions* in the included studies were education (n = 39), training (n = 38), environmental restructuring (n = 38) and enablement (n = 36). The most common *policy categories* were guidelines (n = 39), service provision (n = 38) and environmental/social planning (n = 38). Coercion *(intervention function),* legislation and fiscal measures (both *policy categories)* were not identified in any of the included articles. It was observed that modelling may have been utilized alongside enablement, but it was often difficult to ascertain due to the limited intervention details.

Across the included studies, the following five themes were identified for the factors that influenced the implementation and sustainability of responsive feeding in child care environments: (1) having pre-existing nutrition policies, standards or guidelines in place; (2) education and training associated with the intervention; (3) the effect of providers’ beliefs and confidence in responsive feeding; (4) partnership development and stakeholder engagement; and (5) availability of resources. Each theme and its supporting evidence are described in detail below. Table 3 provides a summary and description of the themes and subthemes.

### 3.1. Theme 1: Existence of Nutrition Policies, Standards or Guidelines

About half of the included studies (n = 18) referenced how the existence of nutrition policies, standards or guidelines impacted the implementation and sustainability of responsive feeding practices. Oftentimes, interventions were implemented as part of funded programs or initiatives, such as being part of the Child and Adult Food program (CACFP) or the Head Start program, which have existing nutrition guidelines and, often, funding to support the implementation of responsive feeding practices [17,37,38,39,40,41,42,43,44,45,46,47,48]. Although some studies discussed the difficulty of existing nutrition policies and guidelines not being enforced [45], others found that the implementation of the intervention provided centres with the opportunity to review their own policies, which had not regularly occurred prior to the intervention as a result of competing priorities [49]. Furthermore, Devine et al. [50] reported that a lack of policy can be a barrier to implementing a healthy eating environment, in general.

Many studies reported how *existing guidelines positively affected* responsive feeding practices. For example, in one study the intervention filled a gap between the existing guidelines and current practice [51]. In five studies, centres who were participating in the CACFP were more likely to adhere to child feeding regulations and to be engaged in responsive feeding practices, such as sitting with children during their meals, in comparison to centres who were not affiliated with the CACFP [17,37,43,44,47]. Another study found that childcare centres affiliated with school districts that follow federal and state nutrition guidelines in addition to district policies, had implemented more nutrition standards, compared to unaffiliated centres [52]. This same study reported on the importance of written policies as a means of sustaining responsive feeding [52]. Alkon et al. [53] noted that campaigns and legislation at the national level that promoted nutrition and physical activity during the time of their intervention may have had a broader positive impact among control centres. There were two studies from the same broad research context that found *misalignment with existing guidelines*. For example, a family-style meal service was noted as a significant challenge in one study and was perceived to violate the guidelines associated with the CACFP [39]. In another study, certain multilevel policies were found to be a barrier in the intervention as they restricted certain hands-on aspects of their intervention, such as obtaining food for cooking projects [40]. To overcome this challenge, providers developed various tactics to work around these specific policies and regulations, which were limiting their activities [40].

### 3.2. Theme 2: Education and Training Associated with the Intervention

The delivery of nutrition education and training related to responsive feeding practices was identified as important for successful implementation and sustainability (n = 17). In particular, training that was effectively designed and delivered had a positive impact on providers’ ability to implement appropriate child feeding and nutrition-related practices. Several studies found that the *place and timing of training delivery* was important for how well the intervention was received [49,50,54]. For example, one study suggested that the online delivery of training was an important factor for maximizing the use of the training [50] and another study suggested that training outside of a normal working day did not work for all childcare centres involved in their intervention [49]. Another study suggested that interventions that include teacher training should not be executed too quickly, they must account for adequate time for educators to internalize the information and incorporate the newly learned practices into their regular practices [54]. Several studies noted the significance of the *qualifications of the individuals* delivering the training and those involved with receiving the training for the intervention [42,55,56,57,58,59]. Two studies noted that it was important to have a qualified educator deliver the training, as they can often assist providers with adopting responsive feeding practices, such as serving meals family-style and sitting with children during mealtimes [42,59]. Farewell et al. [56] found success in leveraging childcare inspectors to deliver education to childcare centres, as they had ongoing relationships with childcare providers prior to the intervention and, therefore, had the potential to positively affect the attitudes and practices of the childcare community. Furthermore, one study noted that the perceived credibility and qualifications of those who delivered the training was more important than the frequency of the training and resulted in greater uptake and positive changes in practices [59]. Specifically, having registered dietitians deliver the training was found to be helpful for improving the practices related to nutrition [55,57], with one study noting the positive difference in the intervention results between their interventions that used a registered dietitian to deliver nutrition education and training, compared to other studies that used different health professionals to deliver nutrition education and training [55]. Other studies emphasized the importance of the *target population of the training*, with several studies noting the importance of training directors or the managers of childcare programs, given that their leadership is essential for supporting other staff with responsive feeding [57,58]. Sigman-Grant et al. [59] suggested that all staff involved in child feeding should be involved in training for a supportive feeding environment.

Some studies spoke to the importance of *ongoing education and training* to sustain responsive feeding practices and support the sustainability of the intervention [45,59,60,61]. For example, one study suggested that, while positive short-term outcomes were found in an intervention, additional training was needed to support the self-efficacy of providers to ensure that the positive outcomes were sustained [61]. Another study found that a short-term intervention, involving a one-day training session for several childcare staff members as a main component, may not have been enough to produce significant positive outcomes, and that a longer or more intense intervention may be required to produce meaningful change [36]. Several studies were associated with long-term initiatives, such as the Child and Adult Care Food program (CACFP) [47,59] or the Head Start program [58], which provided the opportunity to engage staff in ongoing education and training. However, Sigman-Grant et al. [59] found that not all CACFP-funded centres in their study were trained in supportive feeding practices. They suggested that annual, mandatory training for all those involved in child feeding would improve knowledge about the importance of nutrition and child development and would facilitate improved feeding practices. Brewer and Rieg [60] further suggested that greater access to the expertise of a nutrition professional was needed to sustain the positive outcomes of the intervention [60]. Finally, a lack of training and professional development was cited as a limiting factor that impacted the success of responsive feeding in one study [45].

### 3.3. Theme 3: Provider Beliefs and Confidence in Responsive Feeding

Many of the articles (n = 13) discussed the importance of providers’ beliefs in relation to nutrition and responsive feeding practices in childcare, and their confidence in supporting these practices. Examples of providers’ beliefs were related to a reluctance to feed children foods they did not initially enjoy [60], beliefs about the perceived resource-intensiveness of responsive feeding practices [39], the benefits [40] and misconceptions about feeding practices [46] and beliefs about the intervention design (such as interesting topics offered, clear objectives and effective teaching methods) [62].

*Provider beliefs* were found to influence feeding practices in several studies. For example, some studies found that staffs’ beliefs and motivation related to supporting children’s feeding, nutrition and healthy eating environments improved following professional development and workshops [49,50]. In another study, the staff members’ beliefs in the benefits of role modelling behaviors impacted their efforts to engage in this practice [63]. In contrast, many studies found that staff engagement in responsive feeding practices was impeded by their beliefs. Dev [64] reported that providers found mealtimes stressful, which they perceived was a barrier to implementing certain responsive feeding practices, such as difficulty in modelling (eating the same foods as the children or sitting with them to eat), using neutral prompts around mealtimes and children’s reluctance to taste some foods. Another study reported the reluctance of providers to serve new foods after the initial refusal from children [60]. This reluctance appeared after receiving nutrition education about the importance of multiple exposures to new foods, demonstrating how their beliefs continued to influence their feeding practices. A similar hesitancy was found in relation to family-style feeding practices, where providers described various challenges, including time constraints, food wastage and mess, which portrayed their beliefs about the impracticalities associated with allowing children to serve their own food [39,41]. Dev [40] found that providers felt that different responsive feeding practices delivered through nutrition education were important because they encouraged children to learn about nutrition, to try new foods and to promote exploration, however, they described restrictive policies as a barrier to delivering these practices. Two studies spoke to the importance of considering providers’ beliefs in the design of the intervention, as these beliefs can be addressed through the intervention and it can facilitate the implementation of responsive feeding practices [46,62].

*Providers’ confidence* in their knowledge about child feeding and nutrition and their abilities to use responsive feeding practices and support a healthy eating environment was also noted as essential in multiple articles [38,46,50,56,61]. Lanigan [46] described the importance of provider confidence in their gain of nutrition-related knowledge, as this improved their efforts to communicate with families about positive child feeding and healthy eating, in general. One study found success in improving staff confidence to support children’s healthy eating and to discuss this topic with parents through professional development and curriculum modules [50]. Another found that participating in strategic planning improved staff confidence in implementing policy, system and environment changes, including various responsive feeding practices in their childcare setting [56].

### 3.4. Theme 4: Partnership Development and Stakeholder Engagement

The importance of partnership development and stakeholder engagement in the implementation of the responsive feeding intervention program or initiative was identified in many studies as a key factor affecting implementation and sustainability (n = 16). Stakeholders included the institutions, community partners and families of the children who were involved in the intervention of interest.

*Institutional and community stakeholders* were engaged in different ways, for example, in designing and evaluating the intervention for short- and long-term outcomes [38,52,65] and building connections through local infrastructure and institutions [42,62]. Parsons [66] et al. highlighted the value of investing time and resources into building partnerships and gaining buy-in from the relevant community stakeholders for the success of the intervention. Other studies reported that stakeholder engagement helped to guide the implementation of their intervention, such as through the inclusion of an advisory group [65], using a collaborative designing process [38] or by providing resources [67] or program support [40]. In one study, partners were valued child health professionals, who were described as important for keeping providers accountable for achieving the goals set as part of the intervention [49].

Collaborating with existing local institutions that offer programming (e.g., local health departments and universities) and support for families in the community was noted as important, particularly for studies that used a multi-level approach to reach various settings where children live, learn and play [52,62]. Drummond et al. [42] reported success in the sustainability of their intervention as a result of the engagement of stakeholders in the design of their intervention and considered this a critical success factor. They also found that building on local infrastructure and partnering with the childcare community was helpful for the success of their intervention.

*The involvement of families* was also noted as a facilitating factor in the implementation and sustainability of responsive feeding environments [51,57,66,68]. One study specifically stated that the involvement of families in the intervention was important for the sustainability of policy, system and environment changes, including various responsive feeding practices [66].

However, many studies reported that family engagement was a challenge, and that further research could help to identify the strategies that are most effective [50,51,57,68,69]. Brand et al. [38] reported a challenge in responsive feeding practices, which was attributed to a perceived lack of willingness from parents to participate and engage in health-related activities at the centres. A low willingness to participate was noted as parents were invited to take part in cooking classes, for example, but often declined involvement in these activities. In addition, the transfer of nutritional information to the home environment [38] was perceived as lacking. Interestingly, in one study where parents were not heavily engaged in the intervention, they were seen as barriers to the implementation of the intervention [63]. Vaughn et al. [69] emphasized that an important lesson learned from their study was the need for more effective parent-engagement strategies and suggested that using two-way communication instead of passive communication techniques could have been a more effective way of engaging parents. Another study also mentioned a lack of knowledge regarding how to effectively engage parents as a challenge for their intervention, and that the parents were needed for the sustainability of the intervention at home [70].

### 3.5. Theme 5: Availability of Resources

The availability of resources, or lack thereof, was considered an influencing factor for the successful implementation and sustainability of many responsive feeding interventions (n = 16). First, having *financial resources* was noted as being important for making positive changes related to responsive feeding, such as the provision of funding to ensure necessary resources within the respective childcare centres [52,62]. Studies also reported that a lack of financial resources, or a perceived lack of financial resources, was identified as a barrier in the implementation of the intervention [40] and limited the outreach to families [45]. In one study, having childcare centres cover the cost of providers’ meals so that they could engage in responsive feeding practices was considered a barrier to implementing these practices [64].

There were also studies that referred to the effectiveness of *tangible resources and materials,* such as books and online printable forms, in supporting the delivery of nutrition education to children [57], and for facilitating conversations with families about the intervention components [56]. Similarly, in another study curriculum materials were well received by educators for communicating nutrition information, and more were requested [63]. Physical environmental resources, in another study, allowed for responsive feeding changes such as family-style dining [40]. Physical environmental changes were resources such as books, posters, mealtime conversations, hands-on learning and sensory (smell/taste/touch) food exploration [40]. Another study reported that the provision of resources in the form of healthy foods from a food garden and the local food bank facilitated the opportunity for responsive feeding, through nutritionally appropriate meals prepared by the center’s cook and delivered family-style by the educators [67]. In contrast, Vaughn et al. [69] reported that they did not find success in using tangible resources and materials, such as magazines with educational material and at-home activities, to engage families in the intervention, and that other parent engagement strategies may have been more helpful.

Furthermore, the importance of having *sufficient time* to achieve desirable results was discussed in the studies, especially in low-resourced childcare settings [71]. In one study, it was found that staff being given “permission” to spend time focusing on the intervention was important for its success, and keeping this time dedicated to the intervention was important for the sustainability of the positive changes of the intervention [49]. Dev [64] found that a barrier to the implementation of their intervention was a lack of time. In another study exploring the barriers and supports for implementing a nutrition and physical activity intervention in a childcare setting, providers noted that they knew funding would end, but reported that they planned to continue to use the knowledge and resources that they had gained during the intervention to sustain their positive results [57]. It was noted that sufficient time allotment through the restructuring of current schedules was critical to achieve intervention success related to sustaining nutrition education, activities and stories after the interventions were completed and the funding had ended [57].

## 4. Discussion

This review identified 39 articles that described varying interventions that supported responsive feeding in childcare centres. Our identified themes referred to components of interventions at a broader, policy system level, as well as those that related to supporting implementation capacity through partnerships and training, and local factors, such as provider beliefs and available resources. To better understand the results of this scoping review, we examined our results in relation to how our identified themes mapped onto the BCW (see Table 2). The COM-B model can be used to plan interventions for behavior change, and to understand behavior change interventions in the context in which the behavior occurs [23] and was used explicitly by one study included in the review, which developed a tailored intervention to support childcare center compliance with nutrition guidelines [36]. Throughout the following paragraphs, the components of the BCW are used to understand how behavior change theory may help to inform the interventions in the included studies.

*Education*, *training* (i.e., workshops and group sessions) and *enablement* (i.e., coaching, technical assistance and goal setting) were identified as *intervention functions* in almost all of the included studies (n = 39, n = 38, n = 36, respectively). Through the COM-B model, this corresponds with a consideration of an individual’s psychological (e.g., knowledge and psychological skills,) and physical capability (e.g., physical skill, strength and stamina) to engage in the activity of focus and includes necessary knowledge and skills [23]. *Capability* was primarily addressed in the included studies in interventions supporting improvements *to psychological capability,* through the delivery of effectively designed nutrition education and training on responsive feeding practices. To increase the intervention implementation success, the place and timing of the training should be considered in relation to the context of the intervention [49,50,54]. The results from this review also confirmed the importance of the qualifications of the individuals delivering the intervention training or education. Specifically, having registered dietitians deliver intervention training was noted to be helpful for supporting the uptake of information and, as a result, improving psychological capability [55,57]. The target population of the training was also important to note, as training leaders in childcare centres, such as directors or managers, were found to be helpful in improving knowledge and skills [57,58]. Having accurate knowledge to engage in the desired behavior was noted as important in another study outside of the childcare context, which used the COM-B model [72]. This study also identified that ongoing education and training can support the sustainability of interventions specifically addressing the *psychological capability* component [36,45,59,60,61].

Although it is clear that addressing psychological capability through inter- and intrapersonal strategies, such as education and training, is a common approach to designing interventions, focusing on this alone is often not enough to shift behavior, and factors beyond these strategies must also be targeted to facilitate behavior change [73]. The *opportunity* component within the COM-B model broadens our consideration of the factors beyond the control of the individual, which makes a behavior possible or prompts behavior and includes *physical opportunity* (e.g., environment, triggers, resources, location, barriers and prompts) and *social opportunity* (e.g., interpersonal influences, social cues and norms and culture) [24]. *Physical opportunity* was evident in the included studies through the importance of the available resources, such as financial [40,52,62,64] and materials, e.g., books and mealtime accessories [40,56,57,63,67]. Furthermore, having adequate time (or opportunity) for intervention delivery was noted across the studies in this review as critical for successful implementation and, in particular, for sustainability [49,57,64,71]. Another study that specifically used the COM-B model in an intervention with young mothers, also found that time, as a *physical opportunity,* was important for building trusting, supportive relationships and wellbeing in this population [74].

*Social* and *physical opportunity* were recognized through broader support from nutrition policies, standards and guidelines, which guided childcare programs. Existing guidelines positively affected responsive feeding practices and the implementation of the responsive feeding intervention when the guidelines aligned with the intervention [17,37,42,43,47,51,52,53]. In contrast, the misalignment of the intervention with existing guidelines resulted in challenges [39,40]. For example, many studies included in this review were from the United States (n = 31), with some participating centres that were involved in CACFP and Head Start [17,37,38,39,40,41,42,43,44,45,46,47,48], who have their own guidelines and regulations regarding feeding practices. When policies that promoted responsive feeding practices (e.g., guidelines set in place by CACFP or Head Start) were already in place, the implementation of RF practices was more common and successful. For example, Erinosho et al. [42] stated that CACFP, which provides nutrition training and education/resources to childcare programs, may have a carryover effect into mealtime practices and policy implementation. In addition, centres enforcing the guidelines about snacks that can be brought in for parties and celebrations initiated healthier options, such as fruit trays, whole grains and salad bars, that were served in a family-style environment [42]. *Guidelines* and *regulations* were identified as *policy categories* in most of the included articles (n = 39 and n = 30, respectively), indicating that affecting *social* and *physical opportunity* through policies, standards and guidelines was a common and effective practice among the included articles.

Stakeholder and partner involvement were also key in influencing *social opportunity* and affecting the interventions’ implementation and sustainability. Stakeholders were involved in the delivery of the interventions through the *service delivery policy category,* which was identified in all the included studies. Institutional and community stakeholders were engaged in the design and evaluation of the intervention [38,52,65] and in building connections through local infrastructure and institutions [42,62], which emerged as critical to the sustainability of key responsive feeding practices. When families were engaged in the study, their involvement facilitated implementation, providing further social opportunity for intervention implementation [51,57,66,68]. Conversely, several studies struggled to find effective ways to engage families in the intervention as a result of parents’ knowledge and attitudes, which posed a challenge for implementation and sustainability [50,51,57,68,69].

Finally, *motivation,* in the context of the COM-B model, can be understood as brain processes that energize and direct behavior, beyond conscious decision making [23]. It includes *reflective motivation* (e.g., beliefs about what is good and bad) and *automatic motivation* (e.g., wants, needs, desires, impulses and reflexes) [24]. In the behavior system of the COM-B model, an individual’s *capability* and *opportunity* can influence their *motivation* to engage in behavior change [23]. In the included studies, providers’ confidence in their abilities to support responsive feeding practices and their beliefs about the importance of responsive feeding affected their motivation to engage in behavior change, through the implementation of interventions. *Automatic motivation* was identified in the included studies through providers’ confidence in their knowledge and ability to engage in responsive feeding practices [38,46,50,56,61]. An example from the ENHANCE project, which promoted ‘‘whole child’’ development, suggests that the intervention contributed to the efficacy of childcare providers and their belief that their efforts related to childcare, feeding and nutrition have long-lasting effects [46]. In another study professional development in responsive feeding provided the childcare providers with the ability to better enforce and implement policies and practices, along with the efficacy to do this. For example, the intervention demonstrated improved food attitudes for health-promoting behaviors from caregivers. Furthermore, significantly higher confidence levels for nutrition knowledge were reported and enhanced beliefs and behavior changes to match a healthy eating environment resulted [50].

*Reflective motivation* was identified in this review, with interventions targeted at providers’ beliefs, such as pre-existing notions related to feeding practices that affected the providers’ engagement in the intervention positively [63] and, more commonly, negatively [39,41,60,64]. In many of the included studies, the provider’s *motivation (and capability)* was impacted by addressing their beliefs and confidence related to children’s feeding, nutrition and healthy eating environments, through professional development and workshops [49,50]. The capability of a provider may synergistically influence their motivation to deploy responsive feeding practices in a child’s food environment, through increased confidence and a belief in the benefits of the approach.

Other *intervention functions* that directly addressed *motivation* were *persuasion* and *incentivization*. *Persuasion* was used as an *intervention function* in many of the included articles (n = 30)*. Persuasion,* in this context, involved using tactics such as words and images to make the desired behavior more or less attractive, for example, by inducing joy or fun [69]. In the included studies, examples of *persuasion* were encouraging the joy of eating, or using the “accountability” of the educators or individuals delivering the intervention to persuade them to engage in the desired behaviors [49,54]. *Incentivization* was also used as an *intervention function* (n = 27), however, usually through an honorarium, funding or compensation, which would end when the intervention ended, suggesting potential implications for the sustainability of the intervention [44,47,52,62,63,69]. Persuasion and incentivization as intervention functions require further exploration, as these tactics may be criticized as methods of changing behavior that are dependent on the context and delivery of the intervention [39,41,42,62].

## 5. Limitations

This review builds upon past reviews on nutrition in childcare [31] by identifying the factors that influence the implementation and sustainability of responsive feeding, applying a behavior change framework. The scoping review methodology offered a rigorous process to map the current literature, through an extensive search strategy, reviewed by a university library’s subject specialist, with the search results screened and charted independently by two, trained team members. However, one limitation of our process was that the broad nature of scoping reviews does not typically include quality assessments [75], therefore, we were not able to comment specifically on the quality of the included studies. It should be noted that the included studies used a variety of research designs and data collection strategies, which, considering the nature of real-world implementation in childcare environments, was expected [76]. Furthermore, we limited our search to upper-middle and high-income countries to bound our search, and the majority of the literature was from the United States, which may have limited transferability, given the variability in the nutrition policy context across countries. Another challenge was related to the lack of intervention descriptions in the included articles, which may have limited our derived themes and our identified elements of the BCW as a result of the absence of details in the published studies. For example, the intervention function, *modeling*, was often difficult to ascertain as there was not enough description to accurately identify what constituted the modelling actions of the people in the interventions. Including additional context for the intervention process, as well as the outcomes in the published literature would help to support the detail provided in future review articles. Finally, although the scope of the review was limited to childcare settings, our findings suggest the importance of family engagement and future research should explore the importance of the interaction between these environments [77].

## 6. Implications for Research and Practice

A responsive feeding environment is a setting that seeks to promote healthy eating by encouraging a respectful and reciprocal relationship to hunger and satiety and by celebrating healthy and nutritious foods in a supportive atmosphere. The findings of this review suggest five overarching and overlapping themes that are described in the current literature, which influence the implementation and sustainability of responsive feeding environments in childcare settings. Capitalizing on stakeholder partnerships, (e.g., parents, local programs and funders) often required significant resources from the childcare setting in relation to time and money but it may be an important investment for the long-term implementation of a responsive feeding environment [38,40,42,65,67]. Partnership establishment across institutions, from both a funding and a human resources perspective, contributed to the overall intervention success [62]. Education and training provided by qualified professionals, often sourced from stakeholder and partnership development, was critical in the successful implementation of responsive feeding practices, and was particularly helpful for sustaining some of the more difficult responsive feeding behaviors [45,59,60,61]. The place and timing of training delivery (e.g., online or outside of the working day) was related to how well the training was received [49,50,54]. Allowing training sessions to be adequately timed gave educators the opportunity to internalize and incorporate the practices learned [54]. In addition, ongoing education was important in sustaining responsive feeding practices, rather than short-term or sporadic training, which may not be enough to produce sustainable outcomes.

Possibly one of the most important, yet often challenging factors relating to the implementation of a responsive feeding environment concerned educator/providers’ beliefs and confidence. Despite having the *psychological capacity* through education and knowledge training, some studies suggested that providers continued to find mealtimes stressful and were reluctant to engage with certain responsive feeding practices around serving new foods [40,46,49,61]. As noted in one study, the educators’ confidence did not improve during the intervention, which underscores the challenges identified in the literature around shifting educator/providers’ beliefs related to feeding practices [61]. While less is known about providers’ beliefs and confidence with regards to the sustainability of these practices, responsive feeding behaviors are often rooted in personal beliefs and experiences and, therefore, understanding and including them as part of the design or the creation of an initiative was a noteworthy factor in increasing the opportunity for the long-term success of a program [17,39,50,61].

## 7. Conclusions

Our identified themes referred to the components of interventions at a broader, system-level nutrition policy, as well as those that related to supporting implementation capacity through partnerships and training, and local factors, such as providers’ beliefs and the available resources. To better understand the results of this scoping review, we examined our results in relation to how our identified themes mapped onto the BCW and used the COM-B model to understand the factors related to responsive feeding environments that are unique to childcare settings. The policies and guidelines for healthy eating in childcare environments set the tone and supports sustainability but are not enough on their own to support implementation. There is a need to support “actors” or educators to build capability, opportunity and motivation to support behavior change. Only one study specifically utilized a behavior change theory in a targeted way to select their intervention strategies [36], although half indicated they were informed by a theoretical framework. In other studies, the use of theory in the design of interventions was not described comprehensively, suggesting an important future area of study. Through the description of the interventions, it appears that the multiple and overlapping intervention functions of the BCW can support sustainable behavior change in responsive feeding practices in the childcare environment. Some intervention functions outlined in the BCW framework, including restrictions, were used less frequently in the included literature and the practice of coercion was not documented at all in this review. While highlighted in the COM-B as possible paths to behavior change, enforcing restrictions in a childcare environment or utilizing coercive methods to create change in this setting may be more challenging than other, more well-utilized intervention functions, such as education, training or environmental restructuring. Regularly targeting population, community and individual levels through multicomponent, multilevel methods tends to be the most effective way to achieve behavior change [22,24].

## Figures and Tables

**Table 1:** Inclusion and exclusion criteria for the review.

Inclusion Criteria	Exclusion Criteria
Peer-reviewed, English language, primary studies Implementation and sustainability of intervention to support responsive food environments (specific set of activities designed to put into practice an activity or program for responsive feeding) Intervention occurs in childcare centre Upper-middle- and high-income countries Published in the last 12 years (Jan 2009–February 2021) 0–6 years English language	Not peer-reviewed primary study (including protocol and review studies), not English language Not an implementation study Intervention does not focus on responsive feeding practices No process or implementation factors—only about impact on children Intervention is focused on a specific population, which is not generalizable (e.g., children with a specific disability) Intervention is focused on the home environment or other setting outside childcare centre Low-income, lower-middle-income countries Published before 2009 Prenatal period, >6 years old

**Table 2:** Number of Studies, Including Intervention Functions and Policy Categories [23].

Intervention Functions	Definition	Number of Studies Identified Yes (Uncertain)
Education	Increasing knowledge and understanding by informing, explaining, showing and providing feedback through facts.	39 (0)
Training	Opportunities to acquire new skills—physical, cognitive, emotional and social skills—by repeated practice and feedback.	38 (0)
Enablement	Providing support to improve ability to change in a variety of ways not covered by other intervention types.	36 (0)
Incentivisation	Changing the attractiveness of a behavior by creating an expectation of reward or avoidance of undesired outcome, which could be financial, material or social.	27 (1)
Environmental Restructuring	Constraining or promoting behavior by shaping the physical environment (e.g., layout, infrastructure, barriers or equipment) or social environment (e.g., interactions, communication and social support structures).	38 (0)
Persuasion	Using words and images to change the way people feel by making the behavior more attractive (e.g., inducing joy, fun and amusement) or less attractive (e.g., inducing fear, shame or embarrassment).	30 (1)
Modelling	Examples for people to aspire to or copy, as a way of learning and feeling motivated to engage in behaviors.	2 (32)
Coercion	Changing the attractiveness of a behavior by creating expectation of undesired outcome or denial of desired outcomes, e.g., pricing, fines or sanctions.	0 (0)
Restrictions	Using rules to reduce opportunities to engage in a behavior (e.g., bans).	19 (0)
**Policy Categories**	**Definition**	**Number of Studies Identified Yes** **(Uncertain)**
Regulations	Establishing rules or principles of behavior or practice.	30 (0)
Service Provision	Delivering a service;:provision of services, materials and/or social resources and aids.	38 (0)
Guidelines	Creating documents that recommend or mandate practice. This includes all changes to service provision. Documents that make evidence-based recommendations for practice.	39 (0)
Environmental/social planning	Designing and/or controlling the physical or social environment. Architecture, urban and rural planning, object and location design, social care, employment, equality, benefits, security and education.	38 (0)
Communication/marketing	Using print, electronic, telephonic or broadcast media. Mass or digital media campaigns and correspondence.	21 (2)
Fiscal Measures	Using the tax system to reduce or increase the financial cost.	0 (0)
Legislation	Establishing rules or principles of behavior or practice.	0 (0)

**Table 3:** Descriptions of Themes and Subthemes.

Identified Theme	Description	Subthemes	Potential Alignment with Behavior Change Wheel
**Existence of Nutrition Policies, Standards or Guidelines**	Impact of previous or ongoing initiatives on responsive feeding practices.	Existing guidelines positively affected practices and misalignment with existing guidelines.	Focuses on policy-level guidelines IF: restrictions and environmental restructuring
**Education and Training Associated with the Intervention**	Sharing of nutrition information with childcare providers, with the intention of increasing knowledge and behaviors related to responsive feeding.	Time and place of training delivery, qualifications of individuals delivering training, target population of training and ongoing education and training.	Focuses on educator capability IF: education, training and enablement
**Providers’ Beliefs and Confidence in Responsive Feeding**	How childcare provider perceptions of responsive feeding practices affected their willingness or ability to implement these practices.	Providers’ beliefs and provider confidence	Focuses on educator motivation IF: persuasion
**Partnership Development and Stakeholder Engagement**	Individuals who were involved in some capacity of the intervention.	Institutional and community stakeholdersand involvement of families.	Focuses on educatoropportunity IF: enablement
**Availability of Resources**	Importance of having tangible materials as well as monetary support to facilitate implementation or sustainability.	Financial resources, tangible resources and materials and sufficient time.	Focuses on educator opportunity IF: enablement and environmental restructuring

IF—refers to the intervention function from the behavior change wheel theory.

## Supplementary Materials

The following supporting information can be downloaded at: https://www.mdpi.com/article/10.3390/ijerph191911870/s1. Table S1 is included in the submission, which includes a tabular summary of the key characteristics of the included studies. References [78,79] are cited in the supplementary materials.
