# Supplementary material for: Responsive Feeding Environments in Childcare Settings: A Scoping Review of the Factors Influencing Implementation and Sustainability
Source: Int J Environ Res Public Health. 2022 Sep 20;19(19):11870. doi: 10.3390/ijerph191911870 (PMC9564844; doi:10.3390/ijerph191911870)
Supplement: Supplementary file 1 [file ijerph-19-11870-s001.zip › ijerph-1804973-supplementary.pdf]

Author/ year/ location	Major Themes	Research Design	Noted Theoretical Frameworks	Centre Details	Intervention
Agrawal et al. [62] Boston, Massachusetts (USA)	1.Provider beliefs and confidence in responsive feeding<br>2.Partnership development & stakeholder engagement<br>3.Availability of resources	Process-oriented program evaluation - qualitative and quantitative data	1. Social cognitive theory<br>2. Social planning and action model	Head Start programs (3 to 5 years)	Healthy Kids Healthy Futures (HKHF): childhood obesity prevention intervention implemented NAP SACC in 4 Head Start programs. Focus on healthy eating, physical activity, and parental engagement to intervene in all the environments children grow in for multilevel design. Programming was offered to all staff and families with children at the Head Start programs.
Alkon et al. [53] California, Connecticut, and North Carolina (USA)	1.Existence of nutrition policies, standards, or guidelines	Randomized control trial - quantitative data. Pre-post NAP SACC evaluation..	n/a	Licensed child care centres serving predominantly low income families of racial and ethnic diversity<br><br>*No centre previously participated in NAP SACC<br><br>No specified age identified	Five, one-hour NAP SACC education workshops over 7 months at centres for child care directors and staff (e.g., providers, cooks, administrators) on: childhood obesity, healthy eating, physical activity, personal health and wellness, and working with families. Pre & post assessment of polices with random Additional monthly on-site consultations, phone or email consultations, and posters/ information sheets on nutrition and physical activities.

Author/ year/ location	Major Themes	Research Design	Noted Theoretical Frameworks	Centre Details	Intervention
Battista et al. [52] North Carolina (USA)	1.Existence of nutrition policies, standards, or guidelines 2.Partnership development & stakeholder 3.Availability of resources	Pre-post evaluation using NAP SACC - quantitative data	n/a	33 child care centres affiliated and unaffiliated in three school districts. Centres were private, non-profit, and/or head start programs.<br><br>Ages 3-5 years	Local health departments used grant money to recruit centres willing to complete all 4 steps of NAP SACC: initial assessment, goal setting, staff workshops/education with 100% attendance, 6 month follow up. Initial goal setting to improve staff knowledge of nutrition and physical activity recommendations. Aim to improve current practice, sustainability and strength of policy where directors could choose 3 areas of focus followed by three 2 hour workshops within first 2 weeks of program.
Bell et al. [55] Adelaide, South Australia, Australia	1.Education and training associated with the intervention	Pre–post cohort study - quantitative data<br><br>Plate waste assessment of dietary intake	n/a	20 long day care centres (LDCC)<br><br>Ages 2-4 years	Start Right – Eat Right (SRER) dietitians conducted 9 hour nutrition training/education sessions followed by analysis of centres current 2 week menu. Nutrition policy, staff practices, mealtime habits, child intake was observed and contrasted to SRER checklist. Dietitians worked to review list and provide resources and feedback until all centres met all SRER criteria.
Benjamin Neelon et al. [78] Boston, Massachusetts (USA)	1.Education and training associated with the intervention	Randomized controlled trial - quantitative data	n/a	32 licensed centres (12 intervention and 14 control centres)<br><br>Ages < 2 years	Baby NAP SACC to improve practice and policed related to nutrition and physical activity using baseline and 6 month follow up score from EPAO. 47 specific behaviours were targeted with a focus on 4 nutrition areas, 3 physical activity areas, and 1 overall environment area. Trained interventionist conducted 2 workshops that helped teach response to infant hunger cues, appropriate bottle/milk feeding practices, and how to encourage infant activity in addition to ongoing technical assistance provided at least monthly.

Author/ year/ location	Major Themes	Research Design	Noted Theoretical Frameworks	Centre Details	Intervention
Benjamin Neelon et al. [65] North Carolina (USA)	1.Partnership development and stakeholder engagement	Cluster randomized controlled trials - quantitative data<br><br>2 interventions: Baby NAP SACC & Keys intervention	Baby NAP SACC:<br>1. Social Cognitive Theory<br>2. Socioecological Framework<br>The Keys intervention:<br>1. Social Cognitive Theory<br>2. Socioecological Framework<br>3. Self-Determination Theory	Child care facilities maintained by the state licensing and administrative agency.<br><br>Baby NAP SACC 4-24 months (child care centre)<br><br>Keys 18 months – 4 years (family child care homes)	The Baby NAP SACC intervention was 6 months and includes three components 1. self-assessment (directors and parents) and selection of areas for improvement, 2. Health coach technical assistance for directors and parents 3. Provider training. The Keys intervention provided 3 workshops over 9 months and included home visits, coaching calls and educational toolkits. Both interventions utilize health coach to improve childcare environment by advancing nutrition and physical activity practices and policy
Blaine et al. [37] Boston, Massachusetts (USA)	1.Existence of nutrition policies, standards, or guidelines	Cross-sectional baseline data from an exploratory pilot intervention of Baby NAP SACC - quantitative data	n/a	32 licensed child care centres with at least 50% of enrolled children identified as racial minorities<br><br>Age < 24 months	Intervention to assess self-reported infant and toddler feeding practices prior to the IOM recommendations to identify characteristics at the individual- and centre-level that were associated with adherence to IOM recommendations. Differences in feeding practices among child care staff caring for infants versus toddlers was also identified in order to identify age-specific training need. 14 feeding practices identified and 6 were used as primary outcomes based on IOM.
Brand et al. [38] Northwestern Germany	1.Existence of nutrition policies, standards, or guidelines<br>2.Provider beliefs and confidence in responsive feeding	Prospective observational study with baseline and 12 month follow-up measurements - quantitative data	n/a	56 kindergartens of 4 different organizing agencies<br><br>Age 3-6	Baseline and 12 month follow up of three intervention study groups (A, B, C). Group A: organizational approach for comprehensive nutrition concepts (standards and guideline) approach with strong top-down policy. Group B: Centre approach, kitchen staff training at the kindergarten level to emphasize individual decision-making of a centre. Group C: control group, centres organized nutrition-related activities individually, no common approach.

Author/ year/ location	Major Themes	Research Design	Noted Theoretical Frameworks	Centre Details	Intervention
	3.Partnership development and stakeholder engagement				
Brewer et al. [60] Northeastern USA	1.Education and training associated with the intervention<br>2.Provider beliefs and confidence in responsive feeding	Explored perceptions of program through interviews and focus groups - qualitative data	Phenomenology	Low income preschools where 50% of children were classified as obese<br><br>No specified age identified	Local grant funded obesity prevention program that trains school employees to better lead health and physical education lessons to enable them to substantially change daily routines to meet 6 goals:<br>1. Hire certified health coach to instruct 60-minute structure activity weekly and create staff lesson plans weekly<br>2. Purchase new PE equipment<br>3. Incorporate nutrition and activity focused learning into curriculum<br>4. 4 yearly free community-based fitness classes<br>5. 4 yearly parent education meetings for nutrition and healthy eating<br>6. Restructure school snack and meals to reduce packaged/processed foods & increase quality

Author/ year/ location	Major Themes	Research Design	Noted Theoretical Frameworks	Centre Details	Intervention
Buscemi et al. [68] Chicago (USA)	1.Partnership development and stakeholder engagement	Randomized controlled trial - quantitative data	Hypothesized path model which is based on Social Cognitive Theory, and the conceptual model adapted from Skouteris and colleagues.	18 preschools<br><br>Ethnically and racially diverse Head Start programs predominantly African-American<br><br>Ages 3-5 years	The intervention curriculum sought to improve diet quality and physical activity, and decrease television viewing. Teacher-delivered weight control intervention was contrasted against teacher-delivered general health curriculum. Intervention delivery occurred twice weekly of themed 20-minute lesson on healthy eating or physical activity and a 20-minute interactive physical activity component. Control classrooms received once weekly “general health” curriculum.
Chaufan et al. [67] Marin County, California (USA)	1.Partnership development and stakeholder engagement<br>2.Availability of resources	Mixed-methods, and cross-sectional pilot study - qualitative and quantitative data	n/a	North Bay Children’s Centre (NBCC): A non-profit organization that provides childcare<br><br>60% qualify for free or reduced lunch under California state food program<br><br>No specified age identified	Implementation of "Garden of Eatin' to promote early exposure to fresh produce via gardening and lessons to influence preference and long term consumption. Structured time in gardens growing, harvesting, and tasting with produce prepared on site and served family style within a centre. Parents are involved in grocery store trips, education of nutrients, and access to excess produce from the garden. Teacher modeling of healthy eating behavior and activities to promote “culture of health”.
de Silva-Sanigorski et al. [61] Geelong, Victoria, Australia	1.Education and training associated with the intervention<br>2. Provider beliefs and confidence in responsive feeding	Cross-sectional, quasi-experimental design - quantitative data	Guided by health promotion principles and the socio-ecological model	Family day care (home based)<br><br>Ages 0 – 5 years	Romp & Chomp was a 4 year community-based obesity prevention intervention that used health promotion activities to educate and involve families. Health promotion activities were classified into capacity & skill development (workshops, promotional tools, allied health professionals support), facilitation (media coverage, integration of policy, structured guidelines), and advocacy (community awareness, presentations, alliances).

Author/ year/ location	Major Themes	Research Design	Noted Theoretical Frameworks	Centre Details	Intervention
Dev et al, [17] Illinois (USA)	1.Existence of nutrition policies, standards, or guidelines	Cross-sectional study - quantitative data	n/a	Childcare contexts (Head Start n=6, Child and Adult Care Food Program n=17 [CACFP], and non-CACFP n=13)<br><br>Ages 2 – 5 years	Focused on how providers are meeting Academy of Nutrition and Dietetics Benchmarks for Nutrition in Child Care. Key areas of focus were time spend sitting with children at meal time, family style serving, nutrition-education opportunities, nutrient balance and variety, and modeling of healthy eating.
Dev et al. [39] Central Illinois (USA)	1.Existence of nutrition policies, standards, or guidelines<br>2.Provider beliefs and confidence in responsive feeding	Examining perspectives of family style meal service through semi structured interviews - qualitative data	n/a	Licensed urban centre-based child care programs (6 Head Start, 11 CACFP, 7 non-CACFP)<br><br>All child care providers were employed full time at a care program and present at lunchtime or minimum of a snack time<br><br>No specified age identified	Differences in family style meal (FSM) practices of Head Start, CACFP, and non-CACFP child care contexts were qualitative assessed using semi-structured interviews. Head Start programs require FSM practice while CACFP recommend FSM practice. Non-CACFP centres have no specific guidelines outside of state licensing requirements. Each context acted as the intervention to which interview data was collected then coded to assess current feeding practices across providers. Differences in family style feeding, perceptions, and the facilitators and barriers of such were addressed.

Author/ year/ location	Major Themes	Research Design	Noted Theoretical Frameworks	Centre Details	Intervention
Dev et al. [40] Central Illinois (USA)	1.Existence of nutrition policies, standards, or guidelines<br>2.Provider beliefs and confidence in responsive feeding<br>3. Partnership development and stakeholder engagement<br>4.Availability of resources	Exploring perspectives of program delivery through individual semi-structured interviews - qualitative data	Used epistemologic assumptions of the realist method	Licensed childcare centres<br><br>Ages 2 – 5	Provider perspectives of nutrition education and practices of Head Start, CACFP, and non-CACFP child care contexts were qualitatively assessed. Each context was considered a different intervention to capture differences in practice of the 18 Academy of Nutrition and Dietetics proposed benchmarks for nutrition education (NE) in child care settings. Benchmarks included were role modeling, serving, family style, self-regulation, mealtime conversations, hands on food activity, nutrition related books, etc. Each context was asked to indicate if they used that practice or not and coding was used to observe differences in nutrition practices across the 3 intervention types.
Dev et al. [41] Nebraska (USA)	1.Existence of nutrition policies, standards, or guidelines<br>2.Provider beliefs and confidence in responsive feeding<br>3. Availability of resources	Pre-post evaluation - quantitative data	n/a	Licensed Family childcare homes (FCCH) enrolled in the Child and Adult Care Food Program CACFP. All eligible to participate in Go NAP SACC.<br><br>Ages 2-5 years	After providers completed the online Go NAP SACC pre-self-assessment, they attended a six-hour training on child and adult obesity, child nutrition, physical activity, personal health and wellness, working with families, and breast-feeding and infant feeding. Following training, a Go NAP SACC trainer reviewed the pre-assessment results with providers, to identify areas for improvement and to set the goals. Trainers assisted the providers as needed to assist them in reaching their identified goals. Lastly, providers completed the Go NAP SACC post self-assessment.

Author/ year/ location	Major Themes	Research Design	Noted Theoretical Frameworks	Centre Details	Intervention
Dev et al. [64] Nebraska (USA)	1.Provider beliefs and confidence in responsive feeding<br>2. Availability of resources	Cross sectional using self-assessment surveys	N/I	3014 licensed child care programs that participate in CACFP in rural and urban Nebraska<br><br>Ages 2 – 5 years	CACFP intervention in childcare centres versus family childcare homes was assessed across rural and urban settings. CACFP aims to provide nutrition best practice guidelines in childcare including serving practice, limiting high fat/fried foods, moderating celebration foods, family style service, and preparation of healthy foods. Barriers and facilitators of CACFP best practice across settings were assessed.
Devine et al. [50] Australia	1.Existence of nutrition policies, standards, or guidelines<br>2.Education and training associated with the intervention<br>3.Provider beliefs and confidence in responsive feeding<br>4.Partnership development and stakeholder engagement<br>5.Availability of resources	Quantitative survey; qualitative open-ended questions; process and impact evaluation	Health Promoting Schools (HPS) framework (Gillies,Dimitrijevich & Lambert, 2011) and the mandated Early Years Learning Framework (EYLF) (Department of Education Employment and Workplace Relations (DEEWR), 2009).	9 long day care centres both community and privately owned<br><br>*Centres did not have to be currently using SNAC to be enrolled<br><br>Ages 2 – 3	SNAC (Supporting Nutrition Australian Childcare) was extended to include SNACPlus, a virtual professional development (PD) program that offers nutrition and food environment resources and curriculum for child care providers. A PD workshop was offered after baseline (both in-person and online options) to introduce participants to the online SNACPlus resources. Resources included nutrition and feeding modules, curriculum, and provider education and were encouraged to be accessed at any time over 8 months.

Author/ year/ location	Major Themes	Research Design	Noted Theoretical Frameworks	Centre Details	Intervention
Drummond et al. [42] Arizona (USA)	1.Existence of nutrition policies, standards, or guidelines<br>2.Education and training associated with the intervention<br>3.Partnership development and stakeholder engagement	Pre-post assessment - quantitative data	Based on Arizona Steps Models:<br>1.CDC community change model<br><br>2. Border Health Strategic Inititce conceptual framework<br><br>3. Social Ecological Model	30 Child care centres in 6 communities (22 not-for-profit, 6 private/for profit, and 2 school-based centres, 17 Head Start programs including 5 migrant and one tribal program)<br><br>No specified age identified	NAP SACC materials were adapted to a series of seven workshops facilitated by the NAP SACC coordinator at each day care centre as part of Steps to a Healthier Yuma County. Workshops that sequentially built off each other over 9 months<br>1. Increasing awareness of child obesity & program recruitment<br>2. Self assessment of best nutrition and physical activity practices<br>3. Centre specific priority needs assessment and action plans to participate in further NAP SACC based workshops<br>4 – 7. NAP SACC based, centre specific adaption, demonstrations, kits, recipes, interactive exercises.
Erinosho et al. [43] Mississippi (USA)	1.Existence of nutrition policies, standards, or guidelines	Cross-sectional study - quantitative data	n/a	Family childcare homes.<br><br>Ages 3 – 5 years	No intervention, cross-sectional descriptive analysis of nutrition and food practices in home child care. The Child and Adult Care Food Program (CACFP) is a federally regulated program that provides funds to states to support the reimbursement of child care programs, including family child care homes, that provide nutritious meals and snacks to lower-income children. EPAO was distributed to CACFP and non CACFP centres before and after a policy update and nutrition practices were assessed.

Author/ year/ location	Major Themes	Research Design	Noted Theoretical Frameworks	Centre Details	Intervention
Eyler et al. [44] Colorado (USA)	1.Existence of nutrition policies, standards, or guidelines	Post-test design	N/I	Licensed full day child care centres<br><br>Ages 0 – 5 years	Self-reported surveys were disturbed via online and by paper mail to eligible centres. Questions focused on nutrition practices informed by licensing regulation such as meal preparations, length of meal time, TV/media use, beverages, and perceptions of practice by caregiver. Survey also included questions of centre and staff demographics, geography, and centre participation in federally funded support. 344 surveys were completed and assess for self-reported adherence to updated Colorado child care nutrition regulations based on centre characteristics.
Farewell et al. [56] Colorado (USA)	1.Education and training associated with the intervention<br>2.Provider beliefs and confidence in responsive feeding<br>3.Availability of resources	Mixed methods<br><br>Both qualitative and quantitative findings for each component of intervention	Socioecological theoretical perspective	36 child care centres/homes<br><br>*Head start centres excluded due to previous COWP training<br><br>Ages 0 – 5	Healthy Child Care Makes a Healthy Start (HCMHS) intervention to improve attitudes, health practices, and wellness culture in childcare based on Culture of Wellness in Preschools (COWP) program and AIM- P (Assess, Identify and Make it happen for Preschools) to improve nutrient intake and physical activity.<br><br>Intervention components:<br>1. Modified AIM-P process for needs assessment and action plan synthesis via trained COW-P researcher who cofacilitated monthly wellness meetings at centres<br>2. Aim to increase the knowledge and awareness of HEAL strategies among local inspectors and therefore increase the knowledge and awareness in child care providers. Five inspectors and one supervisor took part in four 2-hour interactive HEAL (Healthy Eating and Active Living) trainings presented by trained COWP facilitator. Resources and education materials were provided.

Author/ year/ location	Major Themes	Research Design	Noted Theoretical Frameworks	Centre Details	Intervention
					Both components where considered intensive training and assessed over the following 4-5 months to observe changes via informant interviews, inspection interviews, and caregiver perceptions.
Finch et al. [36] Australia	1.Education and training associated with the intervention	Parallel group randomized control trial	Theoretical domains framework (TDF)	Forty-four childcare centres. An additional sample of 26 centres were recruited and randomized to receive a higher intensity intervention<br><br>All centres prepare and provide food on site.<br><br>Ages 0 – 6	Range of barriers to nutrition guideline implementation were identified from literature reviews, semi structured interviews with eligible centre coos, and on-site observation of food service and menu planning. TDF questioners and the Michie behavior change matrix were applied to survey eligible centres and used to address each barrier appropriately. Cooks and managers were offered one full day workshop to target individual centre weaknesses by providing training, written menu feedback, and printed resources.<br><br>Training combined with didactic and interactive application of skills training, healthy menu planning, and group case studies. Training was conducted by early childhood education and health promotion support staff with at least 5 years of experience as a dietitian and/or in obesity prevention.<br>Written menu feedback came from a trained dietitian in accordance to best practice guidelines.<br><br>Printed resources were the respective nutrition guidelines that outline servings of specific foods that should be served. The resources were to be used to aid in menu planning and compliancy checks. Fact sheets, action planning templates, recommended serving size and goal setting resources were also provided.

Author/ year/ location	Major Themes	Research Design	Noted Theoretical Frameworks	Centre Details	Intervention
					A shorter 3 hour workshop was developed for any staff that could not attend the full day training.
Foster et al. [45] Indiana, Kansas, Michigan, North Dakota, Ohio, South Dakota, and Wisconsin (USA)	1.Existence of nutrition policies, standards, or guidelines<br>2.Education and training associated with the intervention<br>3.Availability of resources	Part of a large-scale childhood obesity prevention project; Assessment Tool used to collect data and identify high-priority areas - descriptive analysis of policies - quantitative data	n/a	Childcare centres serving preschool children in low income rural communities (across 7 states)<br><br>No specified age identified	Intervention involved comparison of implementation of nutrition-related policies. The YMCA's Community Healthy Living Index (CHLI) assessments were used in this study to assess opportunities for active living and healthy eating in all sectors of society, including where people live, work, learn, and play. The Early Childhood Program Assessment Tool is a questionnaire within CHLI that was used to asses the physical environment, promotion efforts, and policies that relate to nutrition in 29 low income centres. The data from the surveys were complied to create descriptive statistical analysis that highlighted high-priority intervention needs in regards to the Academy of Nutrition and Dietetics benchmarks for child care centre nutrition and PA policies.

Author/ year/ location	Major Themes	Research Design	Noted Theoretical Frameworks	Centre Details	Intervention
Joseph et al. [71] Connecticut (USA)	1.Availability of resources	Traditional pre-post intervention design - quantitative data	Obesogenic ecological model: recognizes influences of the home and surrounding environments (i.e., schools) on a child's risk for obesity	One main preschool site with 10-20 kids per room<br><br>Ages 3 – 5 years	70 children from one preschool centre where recruited for a short term nutrition education intervention. The study intervention included one unit (''Eat Smart'') from a previously tested, larger program based on the obesogenic ecological model. Nine 30-minute lessons that focused on teaching kids about healthy and unhealthy snacks foods were taught by a dietitian 4-5 days a week for 2 weeks. Each session offered supporting material for the classroom and a newsletter for parents at home.<br>Pre and post assessment of child knowledge and preference was measured by the Preschool Snack Selection tool (PSS). Both pre and post measures also included anthropometric data for BMI scores, and parental-completed demographic and snack preference forms. Snack choice was also assessed through direct observation immediately before interventions and immediately after to assess child choice of healthy versus unhealthy snack foods after intervention in the childcare environment.

Author/ year/ location	Major Themes	Research Design	Noted Theoretical Frameworks	Centre Details	Intervention
Kristiansen et al. [70] Norway	1.Partnership development and stakeholder engagement	Cluster randomized control trial	n/a	73 kindergartens (public or private)<br><br>Ages 3-5 years at baseline	BRA- study aimed to asses and improve vegetable intake and food environment/practices in preschool children using 3 data collection methods over the course of one year using mixed-model analysis . The intervention focused on addressing 4 determinants: availability, accessibility, encouragement, and role modeling (staff & parents). Parents completed a baseline web questionnaire for child vegetable intake. Then a one-day practical kitchen course was offered to introduce the study, rational, and offered hands on cooking that was observed by an investigator. The day was completed with action planning on the 4 determinants individualized to each kindergarten's needs. Materials such as aprons, food related toys/books, and promotional posters and brochures were given to each kindergarten. Each kindergarten had their own staff support action plan development over the following 4 weeks. A virtual support website, private Facebook group, and booster activities were also offered to boost activities and maintain action plan focus over the intervention time. Immediate baseline follow-up used parent web-based surveys to collect data on child vegetable intake in addition to direct observation by researchers within a centre (2 meals during kindergarten day). A secondary follow-up evaluation occurred after 1 year during which time centres had access to online resources.

Author/ year/ location	Major Themes	Research Design	Noted Theoretical Frameworks	Centre Details	Intervention
Langford et al. [49] England	1.Existence of nutrition policies, standards, or guidelines<br>2.Education and training associated with the intervention<br>3.Provider beliefs and confidence in responsive feeding<br>4.Partnership development and stakeholder engagement<br>5.Availability of resources	A feasibility Randomized Control trial	Social cognitive theory; socio ecological framework	12 nurseries that provided at least 1 main meal a day with minimum of 20 children attending at least 12 hours a week<br><br>Ages 2 – 4 years	Four local Health Visitors were trained to act as NAP SACC UK Partners that would review and revise practice and policies of nutrition and physical education in childcare centres. Partners contrasted current practice against 80 items of best practice in a self-assessment format. Partners aimed to set up eight goals to be implemented over 5 months; and provide nurseries with on-going support via emails, phone calls and face-to-face meetings. Each nursery had local experts delivery 2 staff training workshops on physical activity and nutrition. Workshops were observed and recorded for qualitative data. A NAP SACC at home website was accessible for parents and promoted with newsletters, flyers, and mugs. Parents were asked to use the site to complete a 'Healthy Habits' questionnaire and set goals to improve physical activity, nutrition or oral health in their children. Parents received texts and/or emails to provide support, further information or to encourage further goal setting. Semi-structured interviews also captured quantitative data for all NAP SACC partners and nursery staff in addition to a random sample of parents.
Lanigan et al. [46] Washington State University Vancouver	1.Existence of nutrition policies, standards, or guidelines<br>2.Education and training associated with the intervention<br>3.Provider beliefs and confidence	Longitudinal design - quantitative data	Health Belief Model and Transtheoretical Model	45 Childcare settings (Head Start, community-based non-profit, family home child care, and for-profit centres)<br><br>No specified age identified	The ENHANCE project focused on implementing changes that promoted healthful child weight based on practice and beliefs of a child care provider. Survey data collection occurred at baseline prior to ENHANCE and after 1 year of participation. ENHANCE began with a 7-hour Wellness Retreat (3 hours of feeding and nutrition specific training) that included expert seminars on child obesity prevention, child feeding, and physical activity. Resources were provided to help centres develop and implement action plans to improve policy and practice

Author/ year/ location	Major Themes	Research Design	Noted Theoretical Frameworks	Centre Details	Intervention
	in responsive feeding				with the aid of mini grant funding, training, and social networking.
Lebron et al. [63] USA	1.Provider beliefs and confidence in responsive feeding 2.Partnership development and stakeholder engagement 3.Availability of resources	Focus groups - qualitative	n/a	6 Child care centres from Health Caregivers-Healthy Children sites, low resource backgrounds rural and urban<br><br>Ages 2- 5	Healthy Caregivers – Healthy Children (HC2) is a multicomponent child care centre (CCC) based obesity prevention program targeting teachers, parents, and children in English and Spanish. It was a randomized controlled trial healed over 2 years in 24 CCC's. Directors were responsible for administering environmental and policy changes, and CCC teachers disseminated the program in their classrooms and with their student's parents. Monthly dinner education sessions where healthy recipes and other lifestyle information was shared with parents. Following the 2-year HC2 program, six focus groups were conducted at 6 selected centres (one at each centre) and focused questions on participants experience with HC2 training, implementation, and success in their centre.
Lee et al. [51] Houston, Texas & Phoenix, Arizona (USA)	1.Existence of nutrition policies, standards, or guidelines 2.Partnership development and stakeholder engagement	Community-based participatory research (CBPR) approach; also pre-post intervention - quantitative data	RE-AIM framework; CBPR strategy	6 licensed and accredited childcare centres offering full and half daycare to preschool aged children.<br><br>Ages 3 – 5	The SAGE curriculum was implemented in participating centres through 12 one-hour sessions that used the plant lifecycle as a metaphor for human growth. Sessions included songs, games, and interactive learning activities involving garden maintenance and taste tests for children. Research assistants were trained to deliver the intervention and were taught classroom management skills. Weekly newsletters were used to engage parent's at home.

Author/ year/ location	Major Themes	Research Design	Noted Theoretical Frameworks	Centre Details	Intervention
Liu et al. [47] Large Midwestern city and its suburbs (USA)	1.Existence of nutrition policies, standards, or guidelines<br>2.Education and training associated with the intervention	Descriptive cross-sectional survey & random stratified sample - quantitative data	n/a	Licensed centres and family child care homes where children did NOT bring their own meals<br><br>Ages 3 – 5 years	CACFP early learning centers were compared against non-CACFP centers for nutrition, activity, feeding, and environment best practice. CACFP intervention provides best practice policy and guidelines on menus, feeding, activity, nutrition, training, and screen time for centers. CACFP also offers reimbursement for food and supplies to support healthy practice in a positive child care environment. Goal to identify how the CACFP intervention impacts nutrition and activity practices across centers.
Lyn et al. [48] Georgia (USA)	1.Existence of nutrition policies, standards, or guidelines	Pretest-posttest design - quantitative data	n/a	24 low income licensed childcare centres that were not considered a child care or prekindergarten program in an elementary school. All centres were required to be enrolled in the Child and Adult Care Food Program (CACFP) and provided full day programs.<br><br>Ages 2 – 5 years	The program focused on introducing wellness policies to centres by using EPAO to identify current centre practices around nutrition, activity, and staff behavior. Directors and staff participated in 4 group training sessions during the 15 months. Sessions offered 12 model polices, where each centre had to choose 6 to focus on implementing with the help of grant funding and technical assistance. Post observation looked at how well centres changed based on policy goals set.

Author/ year/ location	Major Themes	Research Design	Noted Theoretical Frameworks	Centre Details	Intervention
Lyn et al. [57] Georgia (USA)	1.Education and training associated with the intervention<br>2.Partnership development and stakeholder engagement<br>3. Availability of resources	Explored experiences implementing intervention through interviews - qualitative data	n/a	22 licensed childcare centres offering full day programs and serving low-income children (part of the Child and AdultCare Food Program) and provide full day programs<br><br>Ages 2 – 5 years	The program focused on introducing wellness policies to centres and providing training, technical support, and ongoing assistance to help implementation wellness policies and goals specific to nutritional and physical activity. The program was a 1-year pilot that provided with $2,000 to support improved healthy snacks, education materials, and PA equipment. Centre directors and staff received training on nutrition and PA, menu planning, food safety, and healthy habits consistent with the wellness policies. Upon conclusion of the program, centre directors participated in an in-depth interview on experiences with program and process they used to make nutrition and activity changes in their centres.
Molloy et al. [58] Midlands of Ireland	1.Education and training associated with the intervention	Simple, randomized, parallel-group study - quantitative data	n/a	42 pre-schools offering a full day care service<br><br>No specified age identified	The aim of the project was to develop a nutrition & health evaluation tool for pre-schools, and to determine whether use of this tool, supported by delivery of a specifically developed resource could promote improved food service and nutrition and physical activity practices in the pre-school setting. Pre-schools were allocated into two intervention training groups: a group in which only the manager of each pre-school was trained and a group in which the manager was trained and the staff were also trained. Training was conducted through a meeting with a dietitian, followed by a discussion about the resource, and provision of individualized feedback and suggested improvement strategies.

Author/ year/ location	Major Themes	Research Design	Noted Theoretical Frameworks	Centre Details	Intervention
Parsons et al. [66] Ohio (USA)	1.Partnership development and stakeholder engagement<br>2.Availability of resources	Interviews, focus groups, and expert panel - mixed methods<br><br>Qualitative thematic analysis	Grounded theory approach	Public health and SNAP Education practitioners and community residents with experience implementing healthy eating policy, systems, and environmental strategies (PSEs)<br><br>Urban and rural counties<br><br>No specified age identified	Policy, system, and environmental (PSE) intervention that combined SNAP-ed practitioners and Creating Healthy Communities public health support to healthy childhood environments that include better nutrition messaging an education. The PSE intervention aimed to identify factors facilitating implementation of PSE healthy eating intervention and sustainability using 5 phases:<br><br>Phase I – Interviews & focus groups (SNAP recipients or SNAP eligible or coalition members) occurred.<br>Phase II – 6 main themes identified to develop programs and conceptual framework<br>Phase III – Expert panel to rank and sort themes based on importance<br>Phase IV – Refining themes and remapping, narrowing scope of intervention<br>Phase V - pilot testing and assessment of validity
Schuler et al. [79] Maryland (USA)	1.Education and training associated with the intervention	Cluster randomized control trial	social ecology behaviour change theory	22 low income child care centres participating in CACFP<br><br>Ages 3 – 5 years	Nutrition promoting training course for centre directors built on the social ecology behavior change theory. The training curriculum was implemented by trained educators over 6 3-hour sessions over 6 months on nutrition, feeding, and activity specific topics. Centres were left with a monthly nutrition education lesson and 3 additional lessons that included materials (books, activities) to be taught by centre staff. EPAO was used to measure centre level observations.

Author/ year/ location	Major Themes	Research Design	Noted Theoretical Frameworks	Centre Details	Intervention
Sigman-Grant et al. [59] California, Colorado, Idaho, and Nevada (USA)	1.Education and training associated with the intervention	Comparison of feeding practices between centres using surveys (other comparison) - quantitative data	n/a	Licensed childcare centres (CACFP-funded centres and non-funded centres)<br><br>Ages 18 months to 60 months	The objective of this study was to compare CACFP-funded to similar non-funded centres in terms of staff's and directors' reported feeding practices. CACFP requires yearly training for staff, although these trainings focus more on program integrity issues than on feeding guidance. The program's feeding suggestions deal with a clean and safe setting, family-style service, preparing enough food to meet the needs of all enrolled children, and allowing additional servings.
Sleet et al. [54] Oklahoma (USA)	1.Education and training associated with the intervention<br>2.Availability of resources	Randomized Control trial	Adult learning theory	9 ECE programs within the Osage Nation tribal community<br><br>Ages 2 – 5 years	Teacher focused intervention that aimed to improve responsive feeding practices using 2 interventions. All 9 programs were assigned to 1 of the 2 interventions. Four programs participated in a 1.5-h teacher-focused responsive feeding practice training (TEACHER). Five programs both participated in the responsive feeding practice training (1.5 h) and also received a 3-h training to implement a 15-wk classroom nutrition curriculum (TEACHER + CLASS). The 2 trainings were held within 2 wk of each other.

Author/ year/ location	Major Themes	Research Design	Noted Theoretical Frameworks	Centre Details	Intervention
Vaughn et al. [69] North Carolina (USA)	1.Partnership development and stakeholder engagement<br>2.Availability of resources	Two arm cluster randomized trial	Social Marketing Approach [23] and informed by the Social Ecological Framework [31], Exchange Theory [24], and Social Cognitive Theory [32].	92 childcare centres<br><br>Ages 3-4	The Health Me, Healthy We (HMHW) intervention was an 8-month social marketing intervention delivered at childcare centres that encouraged responsive feeding and healthy physical activity behaviours. 1 3-hour director and teacher training occurred before intervention kickoff. Intervention kick-off invited participants and offered information of the sessions that would occur. 4 6-week units of education were offered where a 2 hour director and teacher training occurred mid way after unit 2. Informal check-ins (generally <60 min) occurred after the first training and at the end of units one and three and allowed the interventionist to deliver program materials, offer technical assistance (e.g., assess progress and challenges, offer advice), and inquire about event planning.
